# Comparing health-related quality of life of employed women and housewives: a cross sectional study from southeast Iran

**DOI:** 10.1186/1472-6874-12-41

**Published:** 2012-11-23

**Authors:** Fatihe Kerman Saravi, Ali Navidian, Shahindokht Navabi Rigi, Ali Montazeri

**Affiliations:** 1 Health Promotion Research Center, Zahedan University of Medical Sciences, Zahedan, Iran; 2 Mental Health Research Group, Health Metrics Research Centre, Iranian Institute for Health Science Research, ACECR, Tehran, Iran

## Abstract

**Background:**

Quality of life differs for different people in different situations and is related to one's self-satisfaction with life. Considering the role of women in family and social health and the specific cultural characteristics of our province, we aimed to compare the quality of life of employed women with housewives in Zahedan, Iran.

**Methods:**

This cross-sectional study was carried out during 2009–2010 in Zahedan, Iran. The sample consisted of 110 housewives and 110 employed women selected randomly from ten health care centers. Health-related quality of life was assessed using the SF-36. Analysis of covariance (ANCOVA) was used to compare quality of life in housewives and employed women while controlling for age, education and income.

**Results:**

The mean (±SD) age of participants was 33.87± 8.95 years. Eighty-eight women (40%) had a university degree with a mean (±SD) official education of 10.8 (±4.9) years. The results indicated that employed women scored higher than housewives in all measures except for physical functioning. The differences were found to be remarkable for vitality, mental health and role emotional. However, after controlling for age, education and family income, none of differences reached significant level.

**Conclusion:**

After controlling for potential confounders, the findings from this study indicated that there were no significant differences in quality of life between employed women and housewives. However, employed women scored higher on the SF-36, especially on the role emotional, vitality, and mental health. The findings suggest that associations exist between some aspects of health-related quality of life and employment. Indeed improving health-related quality of life among housewives seems essential.

## Background

In recent decades the concept of health has been considered more comprehensively, and therefore, more attention has been paid to the integration of the different aspects of health quality in health assessment. Currently, the assessment of health-related quality of life (QoL) is used widely as an outcome of health care system and health care interventions [1]. The World Health Organization Quality of Life Group defines quality of life as ‘individuals' perceptions of their position in life in the context of the culture and value systems in which they live and in relation to their goals, expectations, standards and concerns’ [2,3]. Therefore, quality of life might mean different things to different people and might also be influenced by many factors including age, culture, gender, education, social class, social environment, diseases, and disabilities [4].

It is believed that job is one of the most effective factors on women's quality of life [5]. In fact it is argued that a woman’s level of education and her employment status are expected to be positively related to women’s empowerment and thus affecting her quality of life [6]. Researchers believe that a woman's choice to work out of the house or to be a housewife depends on her economical and social status and her desire to earn money [7]. Current statistical evidence from developing countries suggests that the participation and role of women in these countries has increased in the professions related to education, nursing, and service occupations [8]. The recent national census in Iran indicates that Iranian women's share of work force is 11%, and currently Iran has about 2.1 million employed women and 14 million housewives [9].

According to sociologists, housework or household chores are facilitating factors for creating a comfortable environment for family members, taking care of and rearing children, and providing the family's necessary requirements and needs. Housekeeping is quite different from other occupations because it is a non-paid job that is done in isolation. Household chores are not usually regulated by national laws, and are repetitive and endless [10]. Studies from Turkey and Iran showed that employed women reported higher quality of life score than non-employed women in all aspects of quality of life [11,12]. Considering the effect of women's health on the overall family health and with regard to lack of coordination in shared responsibility of men and women in family, and considering women's employment as a minor role alongside the major role of housekeeping, this study was designed to compare quality of life of the housewives with employed women in Zahedan, the capital city of Iranian southeastern Sistan and Baluchistan Province.

## Methods

### Design and data collection

This cross-sectional study was carried out during 2009–2010 to compare quality of life of housewives and employed women in Zaheden, Iran. A multiple random sampling methodology was used for the selection of women. Initially, the city was divided into five parts (north, south, center, west, and east). Then, two health care centers from each part were randomly selected (a total of 10 health care centers). All married women aged 14 to 45 years attending the centers were eligible to participate in the study. The exclusion criteria were as follows: (a) women with more than three children, (b) women with a history of physical and mental illness, (c) women not living with their husbands at the time of the study (divorced, widowed and separated), (d) obese and pregnant women, and those afflicted by a special mental and psychological crisis at the time of interview.

### Sample size

The sample size used in this study was determined based on a sufficient statistical power (80%) to detect at least 20% differences in quality of life measures between the two study groups at 5% significant level. As such a sample of 100 women for each group was estimated. However, the actual sample recruited for this study was 110 women per each group.

### Study questionnaire

The data were collected using a two-part questionnaire. Part one consisted of items related to women’s demographic characteristics and part two consisted of the Iranian version of Short Form Health Survey (SF-36v1). The SF-36 consists of 36 questions measuring eight dimensions of quality of life. Score on each dimension ranges from 0 to 100. A higher score indicates a better condition. The psychometric properties of the Iranian version of SF-36 are well documented [13].

### Statistical analysis

Descriptive statistics were used to explore the data. Quality of life was compared between employed women and housewives using analysis of covariance (ANCOVA) while controlling for age, education and family income as covariates. The data were analyzed using SPSS version 19.0.

### Ethics

The ethics committee of Zahedan University of Medical Sciences approved the study. A written informed consent was obtained from all participants.

## Results

### Sample characteristics

In all 220 women were studied. The mean (±SD) age of participants was 33.8 ± 8.9 years. Eighty women (36.4%) had a university degree with a mean (±SD) official education of 10.8 ± 4.9 years. The women’s family income ranged from 5,000,000 to 32,000,000 Rials per month (Rial is the unit of Iranian currency and officially 12,260 Rials = 1 US Dollar). Employed women were older than housewives (mean age 34.9 vs. 32.8, P = 0.07), and were better educated (mean education years 13.5 vs. 7.9, P < 0.0001). The results are shown in Table 1.

**Table 1:** Characteristics of the study samples

	**All (n = 220)**	**Employed women (n = 110)**	**Housewives (n = 110)**	
	**No. (%)**	**No. (%)**	**No. (%)**	**P**
**Age**				0.03*
14-23	27 (12.3)	7 (6.4)	20 (18.2)	
24-33	85 (38.6)	43 (39.1)	42 (38.2)	
34-43	71 (32.3)	42 (38.2)	29 (26.4)	
≥ 44	37 (16.8)	18 (16.4)	19 (17.3)	
Mean (SD)	33.8 (8.9)	34.9 (7.8)	32.8 (9.8)	0.07**
**Education**				< 0.0001*
Illiterate/Primary	41 (18.6)	3 (2.7)	38 (34.5)	
Secondary	99 (45.0)	37 (33.6)	62 (56.4)	
Higher	80 (36.4)	70 (63.6)	10 (9.1)	
Mean (SD)	10.7 (4.8)	13.5 (3.1)	7.9 (4.6)	< 0.0001**
**Family income (Rials)**				
Mean (SD)	5,645,909 (4,350,842)	7,912,727 (4,893,889.8)	3,379,091 (1,926,640.6)	< 0.0001**

* Derived from chi-square test.

** Derived from t-test.

### Comparison of QOL between employed women and the housewives

The comparison of the SF-36 scores for the two groups are shown in Table 2. The lowest means in both groups were ascribed to role emotional (role limitations due to emotional problems) and role physical (role limitations due to physical problems. The lowest and highest ratings for the housewives were on the role emotional (mean=45.4, SD =41.3) and physical functioning (mean=83.6, SD=20.4), respectively. Rating for the employed women ranged from a low mean of 54.5 (SD=40.3) for general health perception to a high mean of 81.3 (SD=20.2) for of physical functioning. Overall, employed women scored higher than the housewives on all subscales except for physical performance. However, the results obtained from the analysis of covariance (ANCOA) indicated that there were no significant differences in quality of life between employed women and housewives.

**Table 2:** The quality of life scores among employed women and housewives as measured by the SF-36

	**All (n = 220)**	**Employed women (n = 110)**	**Housewives (n = 110)**		
	**Mean (SD)**	**Mean (SD)**	**Mean (SD)**	P*	P**
Physical functioning	82.5 (20.3)	81.3 (20.2)	83.6 (20.4)	0.39	0.35
Role physical	52.9 (39.7)	54.5 (40.3)	51.3 (39.1)	0.55	0.48
Bodily pain	64.1 (25.7)	65.5 (23.8)	62.7 (27.4)	0.41	0.68
General health	62.4 (19.6)	62.6 (20.3)	62.3 (19.0)	0.91	0.42
Vitality	55.2 (22.9)	59.1 (21.3)	51.4 (23.9)	0.01	0.08
Social functioning	71.3 (20.9)	71.8 (20.6)	70.9 (21.2)	0.74	0.95
Role emotional	50.1 (42.0)	54.8 (42.4)	45.4 (41.3)	0.09	0.10
Mental health	63.1 (20.9)	66.2 (17.9)	60.0 (23.2)	0.02	0.25

* Derived from t-test.

** Derived from analysis of covariance while job was treated as fixed factor and age, education and family income as covariates.

## Discussion

Although not significant, the findings from this study showed that employed women reported better health status than the housewives in all domains of quality of life except for physical functioning. The findings also hghlighted the fact that the differences were more related to psychological health (role emotional, vitality and mental health) rather than physical health. In fact employment status provided a better psychological health for women when it was compared to non-employed women. Unfortunatetly we did not collect the data on type and employment conditions, but it is argued that not all employment conditions could provide health benefits for women. A prospective study of 21290 female registered nurrses found that low job condition, high job demands, and low work related social support were associsiated with poor health statusat at baseline as well as greater functional declines over the four years follow-up period [14]. However, there might be other reasons for non-significant differences in quality of life between employed women and housewives. For example, the rather low power of the current study (small sample size) could be one reason for such findings.

Interestingly the housewives reported better physical functioning compared to the employed women. It is argued that one reason for low physical functioning among employed women might be due to work-related stress that in turn even could predict sick-leave among employed women [15].

The findings from our study were consistent with the results of similar studies conducted in Iran [12,16,17]. For example, a recent study of 710 working mothers and 350 non-working women from Iran on the impact of emplyment on mothers’ health status found that after adjustment for three main explanatory factors (socio-demographic, work and work-related, and social-life context variables) there were no statistically significant differences between working and non-working women in a range of mental and physical health outcome variables. The authors concluded that this might be a result of the counter-balance of the positive and negative factors associated with paid work such as increased stress on one hand and self-esteem on the other [17]. However, the comparison of quality of life scores between our study population and a sample of Iranian female population showed that the mean score for different quality of life domains in our sample was lower than that of those for the Iranian women population except for physical functioning and mental health [13].

The contemporary women fulfill multiple roles: housewife, partner, parent and caregiver to elders, and worker in the labor force. Within the framework of theoretical models of ‘role’, the relationship between employment and women's health status has been addressed by two major approaches: role strain, and role enhancement. The former argues that multiple roles might have negative effects on women’s psychological well-being, while the latter argues that engaging in multiple roles enhances women’s mental well-being [18]. It seems that the findings from the current study are in favor of the role enhancement approach. In fact this approach emphasizes on women's employment as an additive role to their traditional role; and it is considered as a positive matter. Positive effects of women's employment are probably achieved through increased self esteem, higher income and wider social support.

In general there is a long-standing debate that whether housewives or working wives are happier and healthier. The results obtained from a cross-national data from 28 countries using multi-level analyses showed that housewives were slightly happier than wives who work full time. The cross-level interactions between employment status, and social indicators at country levels also indicated that the disadvantage in happiness for full time working wives improved compared to housewives and part-time workers [19]. Perhaps in the future it might be helpful to examine the topic in the context of the work-family enrichment theory. It studies the extent to which experiences in one role improve the quality of life in the other role [20].

### Limitations

This study had some limitations. Firstly it was a cross sectional study and thus making the findings limited. Secondly, there were some confunders for which we did not collect data. For instance we did not collect information on psychological status of the women at the time of completing the questionnaire. Finally, for illiterate women we did collect the data by interviews while for others we used the self-completion method. Thus the results might be influenced by difference in data collection.

## Conclusion

After controlling for potential confounders, the findings from this study indicated that there were no significant differences in quality of life between employed women and housewives. However, employed women scored higher on the SF-36 especially on the role emotional, vitality, and mental health. The findings suggest that perhaps associations exist between the aspects of health-related quality of life and employment. Indeed improving health-related quality of life among housewives seems essential.

## Competing interests

The authors declare that they no competing interests.

## Authors’ contributions

FKS was the main investigator and provided the first draft. AM analyzed the data, reviewed the paper, responded to the reviewers’ comments and wrote the final draft. AN and SHNR helped in writing processes. All authors read and approved the manuscript.

## Pre-publication history

The pre-publication history for this paper can be accessed here:

http://www.biomedcentral.com/1472-6874/12/41/prepub
